# Aerosol transmission in passenger car cabins: Effects of ventilation configuration and driving speed

**DOI:** 10.1063/5.0079555

**Published:** 2022-02-07

**Authors:** Varghese Mathai, Asimanshu Das, Kenneth Breuer

**Affiliations:** 1 Department of Physics, University of Massachusetts, Amherst, Massachusetts 01003, USA; 2 Center for Fluid Mechanics, Brown University, Providence, Rhode Island 02912, USA

## Abstract

Identifying the potential routes of airborne transmission during transportation is of critical importance to limit the spread of the SARS-CoV-2 virus. Here, we numerically solve the Reynolds-averaged Navier–Stokes equations along with the transport equation for a passive scalar in order to study aerosol transmission inside the passenger cabin of an automobile. Extending the previous work on this topic, we explore several driving scenarios including the effects of having the windows fully open, half-open, and one-quarter open, the effect of opening a moon roof, and the scaling of the aerosol transport as a function of vehicle speed. The flow in the passenger cabin is largely driven by the external surface pressure distribution on the vehicle, and the relative concentration of aerosols in the cabin scales inversely with vehicle speed. For the simplified geometry studied here, we find that the half-open windows configuration has almost the same ventilation effectively as the one with the windows fully open. The utility of the moonroof as an effective exit vent for removing the aerosols generated within the cabin space is discussed. Using our results, we propose a “speed–time” map, which gives guidance regarding the relative risk of transmission between *driver* and *passenger* as a function of trip duration and vehicle speed. A few strategies for the removal of airborne contaminants during low-speed driving, or in a situation where the vehicle is stuck in traffic, are suggested.

## I. INTRODUCTION

The ongoing COVID-19 pandemic has revealed the extent of vulnerability of global populations to the highly contagious respiratory pathogen, SARS-CoV-2. Although initially the modes of transmission of the virus were somewhat unclear, recent evidence has shown that they are spread primarily through tiny droplets and aerosols released by infected individuals and transported by the air to the susceptible during social interactions.^1–13^ This mode of transmission has been recognized by national and international agencies including the Centers for Diseases Control and Prevention (CDC) in the USA and the World Health Organization (WHO) alike.^4^ With the aerosol mode of transmission increasingly identified,^5,14–23^ mitigation measures are important as many countries now face the risk of second and third waves of the more transmissible SARS-CoV-2 variants.

When in an indoor setting, an effective measure for mitigating the risks of airborne transmission is to ensure the environment is well-mixed and ventilated.^24–26^ Indoor air quality is often expressed in terms of the number of air changes per hour (ACH), and when in a confined space with multiple occupants, this can be given in terms of the ventilation rate per occupant in the space, with typically recommended value around 10 l/s per person.^27^ This circulation is achieved using air vents, which are often designed to be physically separate (entrance and exit vents). However, in certain circumstances, a single vent can be used for both inlet and exit flows.^28^ If the space can be considered well-mixed, this air exchange rate can facilitate quick dilution of airborne particles, thereby reducing the viral load. Additionally, studies have proposed improved social distancing guidelines while inside indoor spaces, using space–time diagrams^29^ that take into account not just the physical separation (space) between the interacting individuals but also the duration of the interaction (time), which can lead to an increased risk of airborne transmission.^30^

One setting that highlights the risk of airborne diseases transmission in confined spaces relates to travel. There are several factors which make transportation a high-risk environment, most importantly the prolonged duration of exposure to passengers who are not from the same household. Travel-related interactions can contribute, in various degrees, to the spread the SARS-CoV-2 virus. Several documented infections are attributed to airborne transmission.^31–34^ In commercial airplanes, the vertical airflow patterns and the filtration of cabin air every 3 min using high-efficiency particulate air filters (HEPA) potentially lead to a low risk of transmission.^35^ In contrast, the flow patterns in public buses are very different,^36^ and recent studies have highlighted the special precautions needed to reduce the risks of transmission among passengers.^32^ The risk of airborne transmission in passenger cars is quite variable and is strongly dependent on the specific configuration.^37,38^ While the typical passenger car ride hosts fewer occupants, the enclosure volume of a car cabin is also significantly smaller. More importantly, air conditioning systems in cars are typically designed to optimize the comfort of the occupants, not reducing airborne disease transmission, and although HEPA filters are commonly used in modern automobiles, they are of variable quality.

Ott *et al.*^39^ and Saber and Bazargan^40^ studied the persistence of cigarette smoke inside the cabin of a passenger car subject to different ventilation scenarios, while Müller *et al.*^41^ assessed the concentration of contaminants entering from outside the cabin. However, these studies did not examine the microclimate within the cabin or the transport of airborne pathogens from occupant to occupant. To address this, Mathai *et al.*^1^ performed a numerical study of the air exchange rates and aerosol transmission within the cabin of a typical passenger car, quantifying the utility of opening the windows and allowing fresh air to enter the cabin and flush out potentially pathogenic airborne particles. They simulated the case of an automobile driven at a relatively high speed of 50 miles per hour, and explored various combinations of open and closed windows. The air flow around the vehicle establishes an external pressure distribution and it was found that the air typically enters the car through the rear windows and exits the cabin via the front windows. When all four windows were opened, they found a high air exchange rate of 250 ACH, or equivalently, 50 l/s per person. When two windows, namely, the rear-left (RL) and the front-right (FR), were opened, this resulted in an air draft entering the RL window and exiting the FR window with an air change rate of 150 ACH, or 30 l/s per person.

However, there are many practical challenges to driving with windows fully open. For example, during cold winter months (or hot summer months) the strong blast of incoming air along with its buffeting noise can be discomforting to the occupants.^42,43^ Moreover, the ventilation rates computed for fully open windows were well in excess of the typically recommended values in most ventilation guidelines.^27,44^ Therefore, it may be worthwhile to explore partially open windows as a practical compromise when the driving speeds are sufficiently high, and a few alternate ventilation strategies when the driving speeds are low.

**FIG. 1.**
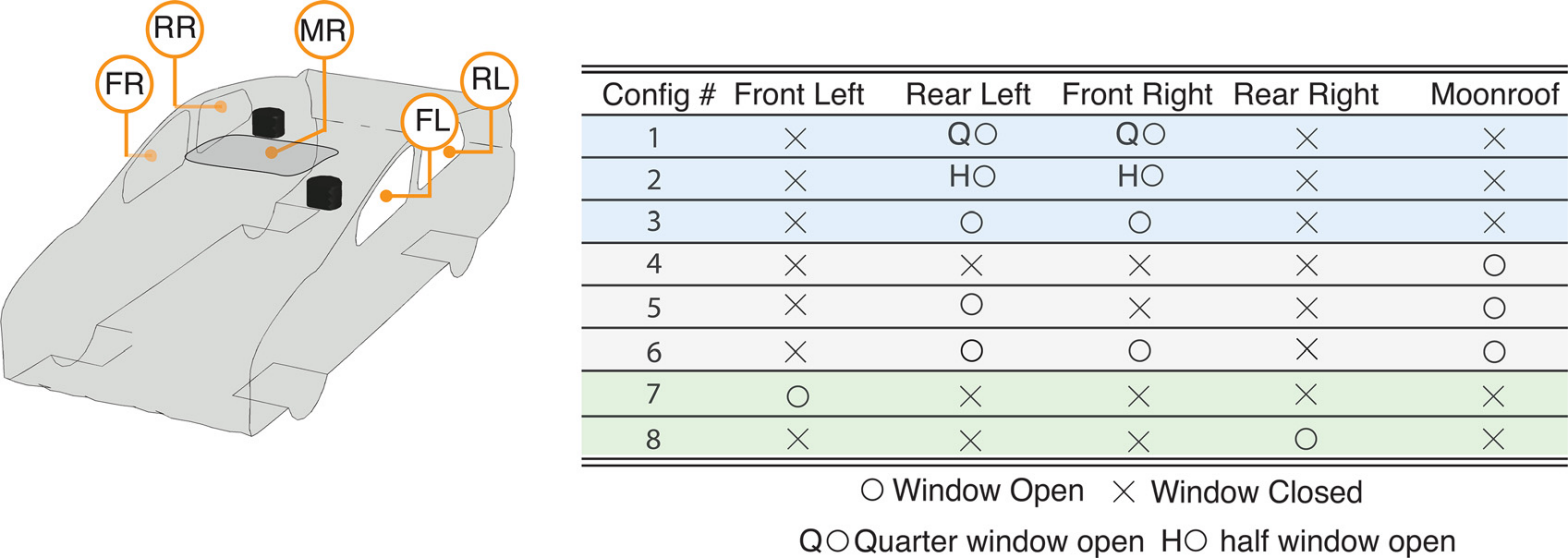
Model car geometry and driving configurations studied in the present work. (a) Schematic of the model car geometry with window identifiers the front-left (FL), rear-left (FL), front-right (FR), rear-right (RR), and moonroof (MR). The two regions colored in black represent the faces of the *driver* and the *passenger*. Table on the right side summarizes the eight configurations simulated, with various combinations of open and closed windows. The blue color shading in the rows (Configs. 1–3) refer to cases where the RL and FR windows were partially or fully open at a driving speed of 50 miles per hour. Rows with gray color shading refer to configurations with an open moonroof (Configs. 4–6) at the same driving speed. Rows with green color shading refer to configurations where the vehicle is at a standstill (Configs. 7 and 8).

In the present work, we continue the approach taken by Mathai *et al.*^1^ and explore an extended range of practical driving configurations, including partially open windows, driving with an open moonroof (MR), and driving over a range of speeds. In Sec. II, we present the details of the computational approach and describe eight driving configurations (Configs. 1–8 in Fig. 1). Recognizing the biological variability and the unknown factors in the infectivity (viral shedding rate) of individuals (factors including stage of infection, anatomy, age, and obesity^45^), our focus here will be to evaluate the “relative” risks of transmission, rather than assigning absolute values to the infection risk. Hence, we will be presenting comparisons of various driving configurations and expressing transmission in terms of “percentages” of released aerosols by one occupant inside the cabin, and reaching the other.

In Sec. III, we begin by analyzing the airflow patterns and aerosol transmission with partially open windows. We then discuss the implications of opening the moonroof and its utility as an exit to the air flow. A few of the configurations studied by Mathai *et al.*^1^ will be revisited for the purpose of drawing comparisons. As a baseline case, we consider driving with all four windows closed and the air-conditioning system turned on—with air entering the cabin at the dashboard and exiting near the rear of the cabin—that is common to many modern automobiles.^1,46^ We evaluate the reduction in air exchange rate as a result of lowering of driving speed and its effect on aerosol transport. Modifying the approach of Yang *et al.*,^29^ a model *speed–time* diagram will be presented, which can be used to assess the relative concentration of aerosols in terms of the driving speed and the ride duration. We then address the situation where the vehicle is stuck in traffic (i.e., at zero velocity) and discuss a few practical strategies for ensuring a good ventilation when the natural flow of air due to the vehicle speed is absent. The main conclusions of the study and future directions are discussed in Sec. IV.

## II. COMPUTATIONAL APPROACH

The simulations were performed to numerically solve the steady-state, three-dimensional Reynolds-averaged Navier–Stokes (RANS) equations. The turbulence model used for closure was the standard k–*ϵ* model. A second-order upwind scheme was used for numerical discretization, with the SIMPLE algorithm^47^ for the pressure–velocity coupling on a finite-volume-based computational fluid dynamics package (Ansys-Fluent v2020). The computational domain was of size 6h×5h×3h in the streamwise, vertical, and spanwise directions, respectively, where *h* is the car height. The car geometry was loosely modeled on a Toyota Prius. The three-dimensional model of the vehicle was created in SolidWorks CAD modeling package and the computational mesh was generated using the Ansys-Workbench meshing tool. The inlet flow boundary condition was a uniform velocity inlet, and a pressure outlet was applied to the exit and tangential velocity prescribed on the sides of the domain. In common with Mathai *et al.*,^1^ the interior of the cabin was simplified, and the *driver* and *passenger* were modeled as two cylindrical bodies. The equations solved were

$${\bar{u}}_{j}\frac{\partial {\bar{u}}_{i}}{\partial x_{j}}=\frac{1}{\rho }\frac{\partial \bar{p}}{\partial x_{i}}+\nu \frac{\partial^{2}{\bar{u}}_{i}}{\partial x_{j}\partial x_{j}}-\frac{\partial \bar{u_{i}^{\prime }u_{j}^{\prime }}}{\partial x_{j}},$$ (1)

where $\bar{u_{i}}$ and $u_{i}^{\prime }$ represent the mean and fluctuating velocity components, respectively, $\bar{p}$ the mean pressure, *ν* the kinematic viscosity, *ρ* the density of air, and *x_i_* the three spatial coordinates and *t* the time. Here, the $\bar{u_{i}^{\prime }u_{j}^{\prime }}$ terms are modeled in terms of the turbulent kinetic energy, *k*, and the turbulent dissipation rate, *ϵ*, obtained using the standard *k*–*ϵ* model.

The dispersal of aerosols within the cabin space and outside are treated as a passive scalar, and the average concentration, ϕ, is given by the species transport equation,

$$\bar{u_{i}}\frac{\partial \phi }{\partial x_{i}}=D\frac{\partial^{2}\phi }{\partial x_{i}^{2}}-\frac{\partial \bar{u_{i}^{\prime }\phi \prime }}{\partial x_{i}},$$ (2)

where ϕ and ϕ′ represent the mean and fluctuation, respectively, of the aerosol concentration, and *D* is the molecular diffusion coefficient. $\bar{u_{i}^{\prime }\phi \prime }$ is expressed in terms of the turbulent diffusivity.^48^ The transport equations were solved until the residuals reduced to less than 1 × 10^−6^. The wall unit $y^{+}$ was chosen to lie in 30–300 range, within the log-layer region.^49–52^ Grid independence analyses were performed using three different incremental grid resolutions, where the boundary layer element size was varied from 0.004 to 0.0008 m. These showed no appreciable difference for the overall pressure, velocity and concentration fields, as well as for the averaged concentration and air-exchange rates.

Note that in our analysis the molecular diffusion term is negligible compared to advection and turbulent diffusion. The scalar is a proxy for non-interacting aerosol released continuously by the occupants. The diffusion of the passive scalar is primarily driven by advection and turbulent diffusion effects. This was validated by injecting various concentrations of the passive-scalar and observing that the scalar field (percentage) within the cabin was unchanged. This approach mimics the mixing of a high Schmidt number species, such as smoke or aerosols released within the cabin.^39^

We note that the RANS approach employed here is not as accurate as direct numerical simulations (DNS) where the full Navier–Stokes equations are solved, resolving all relevant length scales and time scales of the turbulent flow. Similarly, other approaches, such as large eddy simulations (LES), might offer improvements in accuracy compared the Reynolds-averaged approach. However, DNS or LES can become prohibitively expensive computationally and are often impractical for large-scale flows, especially to address the timely subject of airborne transmission where multiple simulations and comparisons are necessary. In this regard, the Reynolds-averaged approach has been used widely in obtaining insights about turbulent flows and recently to study the airborne disease transmission.^53–59^

## III. RESULTS AND DISCUSSION

**FIG. 2.**
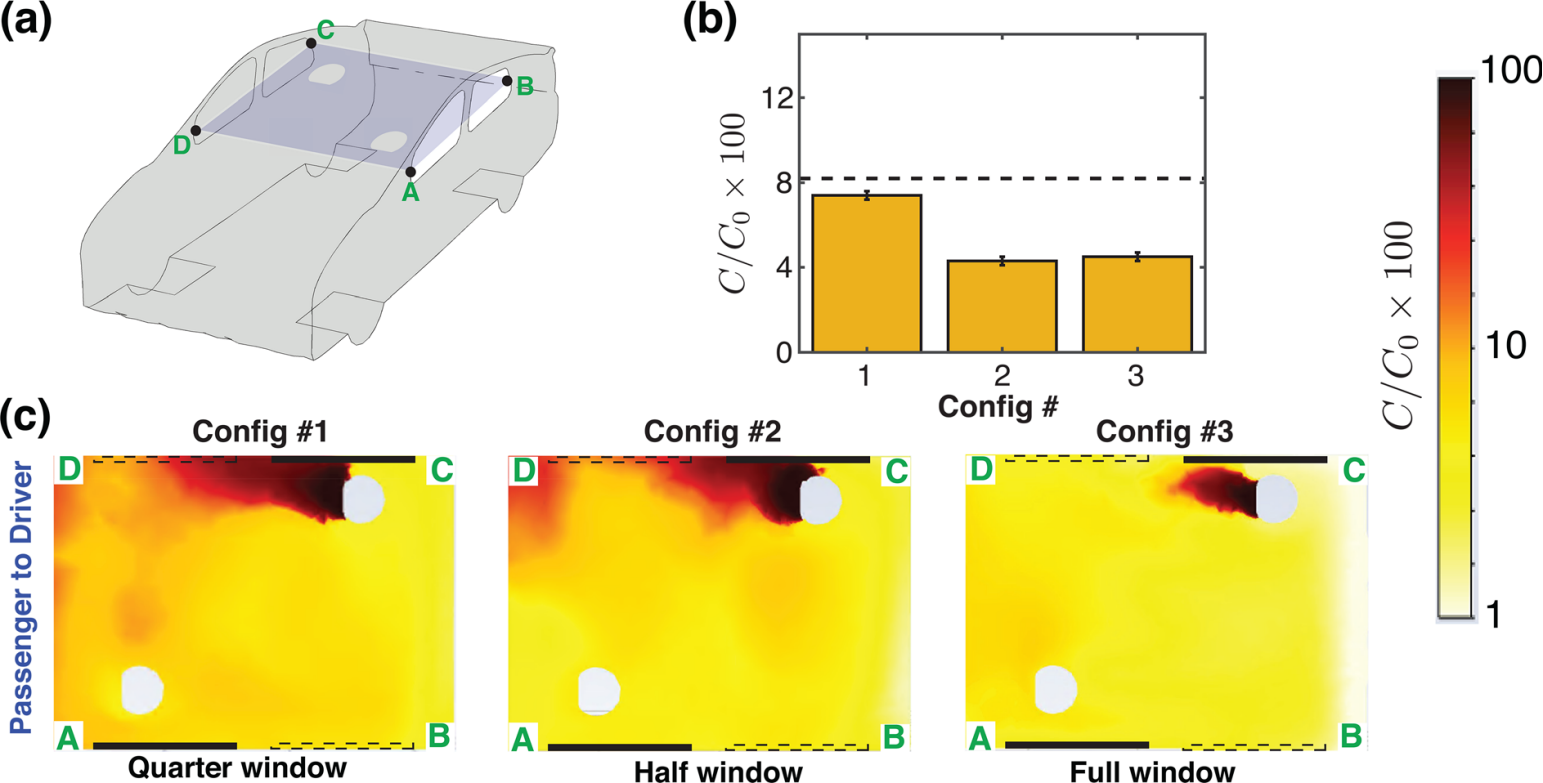
Passenger-to-driver transmission with partially open RL and FR windows. (a) Schematic of the vehicle with a cut plane A–B–C–D passing through the mid-height of the windows. (b) Average concentration of aerosols, originating from the *passenger*, and reaching the *driver*, expressed as a percentage. The dashed black line is the average concentration that reaches from the *passenger* to the *driver* when all windows are closed and air conditioning turned on (Config. 1 of Ref. 1). (c) Concentration field of aerosols originating from the *passenger* for quarter open, half open and full open windows. The dashed and the solid lines overlaid here denote open and closed windows, respectively. Note that the line segment A–D is at the front of the car cabin, so the air flow direction in (c) is from left to right when viewed in the frame of reference of the moving car.

We first consider the case of the vehicle driven at 50 miles per hour and having two windows, rear-left (RL) and front-right (FR), open. It was revealed in prior work^1^ that this configuration resulted in a good cross-ventilation and air exchange rate, well-exceeding the recommended ventilation rates for enclosed spaces.^27^ Since having the windows fully open may not be practical under all weather conditions, we now consider the airflow patterns and aerosol distribution with the windows partially open, i.e., RL and FR windows quarter open (QO—Config. 1), and half open (HO—Config. 2). Following the convention used in Ref. 1, Fig. 2(a) shows a schematic of the car geometry with a horizontal cut-plane passing at the mid-level of the windows. The bar graph [Fig. 2(b)] shows the relative concentration of aerosols, $C/C_{o}$, in a 10-cm-diameter spherical zone surrounding the *driver* for the quarter-open (Config. 1), half-open (Config. 2), and fully open (Config. 3) windows. These values are also summarized in Table I. As a reference, a case with all four windows closed and air conditioning turned on is indicated with the dashed line. In Fig. 2(c), the concentration field on the horizontal midplane A–B–C–D is shown. Recalling Ref. 1, this configuration is characterized by a strong current of air entering at the rear-left (RL) window, flowing across the back of the cabin and over the *passenger* before turning toward the front and exiting the cabin through the front-right (FR) window. We note that the configuration with windows quarter open does not establish a strong air exchange rate and the aerosol concentration experienced by the *driver* [Fig. 2(b)] is very similar to the windows-closed case. However, having the windows half open significantly reduces the aerosol transmission from *passenger* to *driver*, and opening the windows further, to fully open (Config. 3), has only an incremental benefit over the half open configuration.

**TABLE I.** Summary of air exchange rates and aerosol transmission between the occupants (Driver—*D* and Passenger—*P*) for Configs. 1–6, which are at a driving speed of 50 miles per hour. Baseline configuration refers to the case where all windows are closed and the air conditioning system is turned on with fresh air intake near the dashboard and exit vents at the rear end of the cabin.^1^

Config.	Air change rate ( $h^{-1}$)	Ventilation rate (l/s/person)	$C/C_{0}\times$ 100	
			P→D	D→P
Baseline	65	14	8.4	11.2
1	72	15	7.4	13.8
2	96	20	4.3	6.5
3	140	30	4.5	2.5
4	138	29	8.5	4.2
5	211	44	2.1	0.9
6	264	55	1.2	1.6

**FIG. 3.**
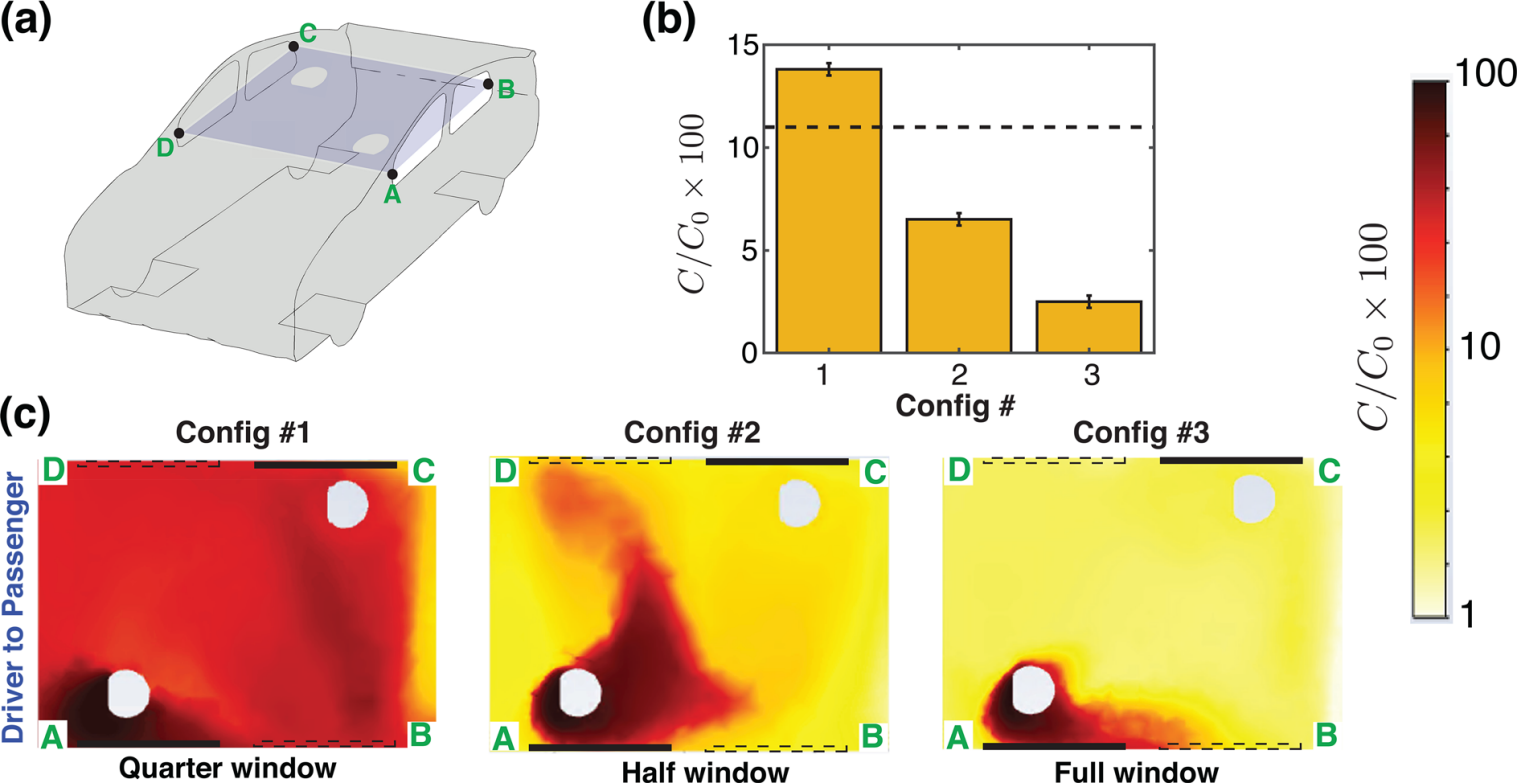
Driver-to-passenger transmission with partially open RL and FR windows. (a) Schematic of the vehicle with a cut plane A–B–C–D passing through the mid-height of the windows. (b) Average concentration of aerosols (expressed as a percentage) that originated from the *driver*, and reaching the *passenger*. The dashed black line is the average concentration that reaches the *passenger* when all windows are closed and air conditioning turned on (Config. 1 of Ref. 1). (c) Concentration field of aerosols originating from the *driver* for quarter open, half open, and full open windows. The dashed and the solid lines overlaid here denote open and closed windows, respectively. Note that the line segment A–D is at the front of the car cabin, so the air flow direction in (c) is from left to right when viewed from the frame of reference of the moving car.

Figure 3 illustrates the transmission from *driver* to *passenger* for partially open and fully open windows (Configs. 1–3). Again, the dashed line [Fig. 3(b)] shows the reference case with all four windows closed and air conditioning with fresh air intake on. Surprisingly, we see that the quarter window open case has a higher driver-to-passenger transmission than the all windows closed case. This occurs because the fresh air intake rate due to the air conditioning (14 l/s per person) is comparable to the air exchange rate for the quarter windows open setting (15 l/s per person). Here again, for driver-to-passenger transmission, the half window open case (Config. 2) performs well, with a noticeably lower concentration of aerosol transmission than the quarter open case. For windows half-open, the concentration of aerosols reaching the *passenger* has reduced to around 6.5%, [Fig. 3(b), Table I]. With the windows fully open, the air exchange rate is further improved, and only about 2.5% of the released aerosols from the *driver* reaches the *passenger*. These results suggest that once a sufficiently strong air flow pattern is established inside the cabin, the aerosols released by either occupant are removed efficiently, and our analysis of partially open windows (Figs. 2 and 3) suggests that having windows half-open may present a practical compromise when driving at a relatively high speed.

**FIG. 4.**
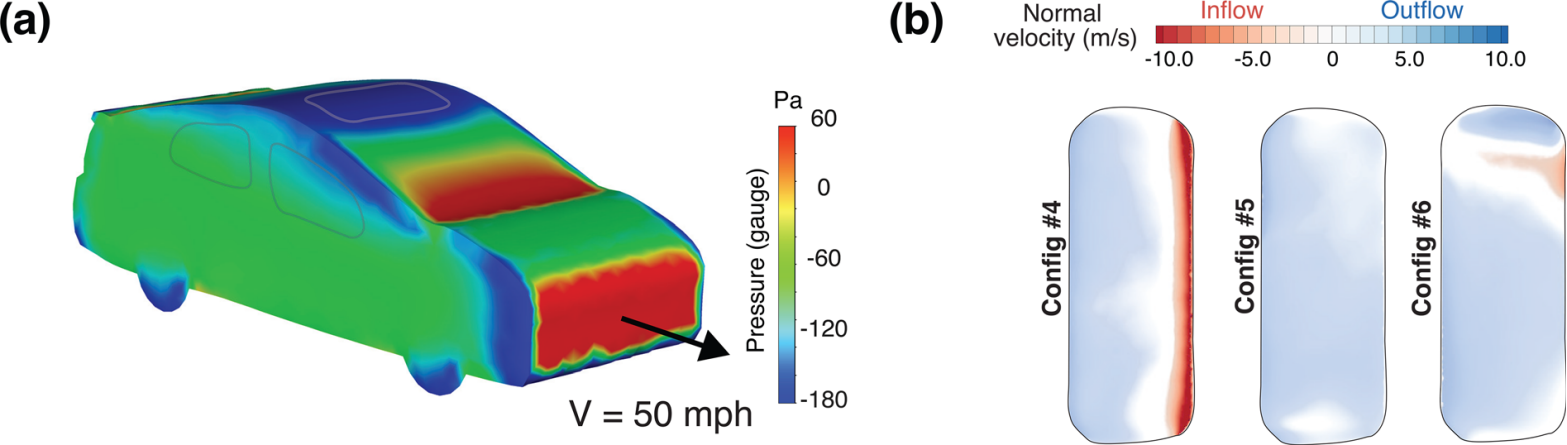
Pressure distribution on the surface of the car and moonroof flow. (a) Gauge pressure on the exterior surface of a car at a driving speed of 22 m/s (50 miles per hour). The color bar shows the gauge pressure in Pascal, and highlights the range [−180,60] Pa. Note that at this driving speed, the full range of gauge pressure on the surface is [−361,301] Pa. The moonroof surface has a lower pressure relative to other regions. (b) Normal velocity on the open moonroof surface for Configs. 4–6. The first case (Config. 4) has only the moonroof open, while all windows on the sides are closed. In Configs. 5 and 6, a cross-ventilation path is established by opening one additional window (MR and RL open, as in Config. 5) and two windows (MR, RL, and FR open, as in Config. 6).

Mathai *et al.*^1^ demonstrated that having at least two windows open establishes an effective cross-ventilation path, and one of the design principles used by the authors to identify the windows to open was based on the pressure distribution on the surface of the car [Fig. 4(a)], which revealed that the side windows on the front had a slightly lower pressure than the side windows on the rear. Consequently, when one rear window and one front window were opened, fresh air predominantly entered the cabin through the rear window, and exited through the front window. From Fig. 4(a), we note, however, that the lowest pressure on the surface of the car lies on the roof of the car where, if installed, the moonroof is located. Applying the same principle to the selection of inlet and exit air paths, one may, therefore, choose the moonroof as one of openings. We explore this concept by studying three configurations (Configs. 4–6) wherein the moonroof (MR) is kept open.

Config. 4 has the moonroof open, while all other windows remain closed (Fig. 1). In Config. 5, the moonroof and the real left (RL) window are opened. This establishes the maximal pressure difference possible, while also ensuring two separate openings. In Config. 6, the moonroof (MR), the front-right (FR) window, and the rear-left (RL) window are opened.

Figure 4(b) shows the normal (inlet/exit) velocity on the moonroof surface viewed from above for Configs. 4–6. The left side of the image denotes the leading edge of the moonroof, and the right side its trailing edge. Regions of the moonroof in blue indicate an outflow from the passenger cabin, while regions in red indicate an inflow into the cabin. When only the moonroof is open (Config. 4), we observe a strong inflow region confined to the rear-edge of the moonroof. Since the net flow is zero (as there is only one opening), this rate of inflow is balanced by a milder outflow that is distributed over the front region of the moonroof. Note that this configuration might, in practice, be accompanied by a strong Helmholtz cavity pulsation due to the compressibility of the air, and would likely be very uncomfortable for the cabin occupants.^60^ In Config. 5, we see that the normal velocity distribution is greatly modified when a rear window (RL, in this case) is opened in addition to the moonroof. The favorable pressure gradient between the RL window and the MR may be thought of as driving this cross-flow, allowing fresh air to enter the cabin through the RL window and exit through the MR. It is interesting to note that the normal velocity distribution on the moonroof is nearly uniform despite the left-right symmetry breaking due to the open RL window. This suggests that the pressure difference driving this flow is dominant. In Config. 6, an additional window (FR) is opened. Since the FR window also has a relatively low pressure [Fig. 4(a)], a fraction of the incoming air from RL window leaves through the FR window. Because the moonroof now competes with the open FR window, the right side of the moonroof sees a region of inflow for Config. 6. The air exchange rates for the three configurations are presented in Table I. When only the MR is open, we obtain an air exchange rate of 138 ACH, or equivalently 29 l/s per person. When the RL window and the MR are both open, the air exchange rate increases dramatically to a value of 211 (44 l/s per person), while for Config. 6, the ACH climbs further to a value of 264, or equivalently 55 l/s per person.

**FIG. 5.**
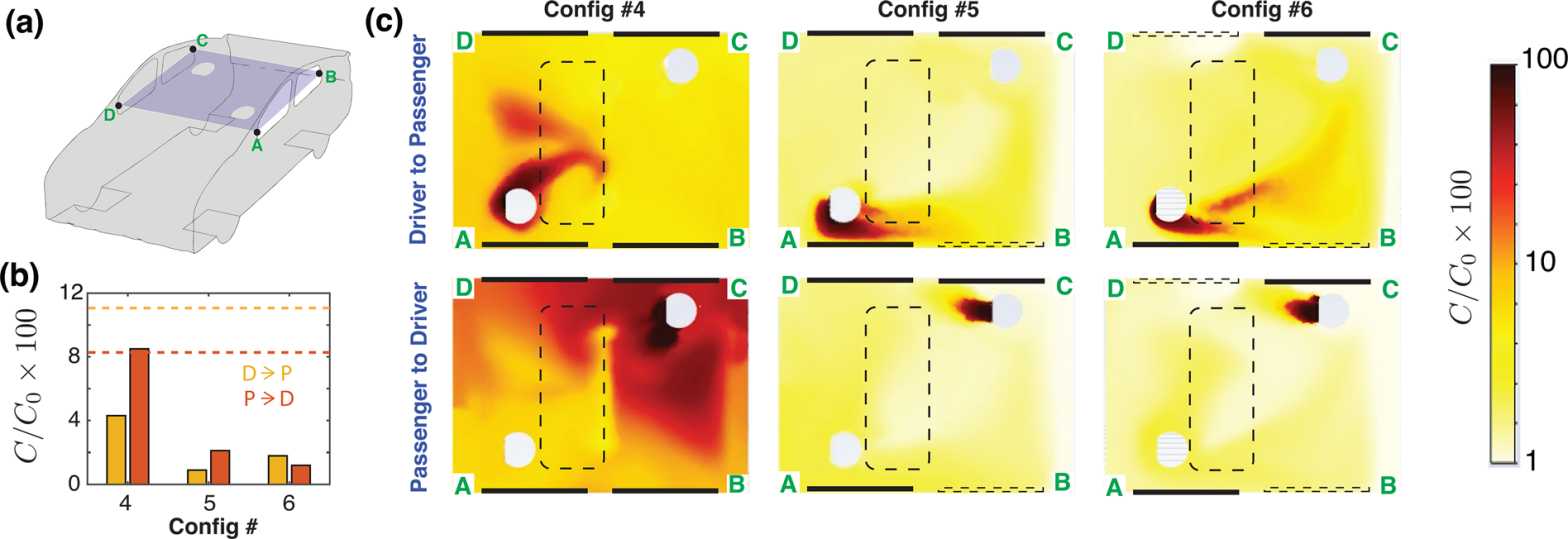
Driver-to-passenger (D → P) and passenger-to-driver (P → D) transmission for a vehicle with a moonroof, driven at 50 miles per hour ( ≈ 22 m/s). (a) Schematic of the vehicle with a cut plane, A–B–C–D, passing through the center of the cabin on which the subsequent concentration fields are shown. (b) Relative concentration of aerosols reaching the occupants for the three configurations with moonroof open (Configs. 4–6). Red bars are the concentration of aerosols near the *passenger*, which originated from the *driver*, and the orange colored bars show the concentration of aerosols near the *driver*, which originated from the *passenger*. (c) Panel showing the aerosol concentration on the mid-plane A–B–C–D for the three configurations with the open moonroof. The upper row of the panel shows the concentration field for the species originating from the *driver*, whereas the lower row shows the concentration field for the species originating from the *passenger*. The dashed and the solid lines denote the open and closed windows, respectively. Note that the line segment A–D is at the front of the car cabin, and the flow direction in (c) is from left to right when viewed in the frame of reference of the moving car. The dashed line denotes an open window and the solid line indicates a closed window. The moonroof facilitates the efficient removal of potentially infectious aerosols from the cabin.

The aerosol transmission between the occupants, for Configs. 4–6, is shown in Fig. 5(b). The dashed lines in orange and red correspond to the reference case where all windows are closed with the air conditioning turned on. Figure 5(c) shows the concentration fields of the aerosols on a plane located at mid-height of the cabin. The upper row shows the transmission from the *driver* to the *passenger* (D → P), while the lower row simulates the case of an infected *passenger* releasing aerosolized particles. The location of the open moonroof is illustrated using a dashed boundary. In general, the proximity of the moonroof to the *driver* allows for some of the aerosols released by the *driver* to be flushed out. The *passenger*, however, is located farther from the moonroof. Therefore, for P → D transmission, the moonroof does not remove the aerosols as effectively as in the D → P case.

With only the MR open (Config. 4), the concentration of aerosols is the cabin remains relatively high ( ≈ 8%), particularly when considering passenger-to-driver transmission (P → D). In comparison, for D → P transmission, the average transmission is lower ( ≈4%) due to the proximity of the *driver* to the MR. The aerosol concentration fields improve significantly when the additional window (RL) is opened [Figs. 5(b) and 5(c)]. The rear window acts as the primary inlet for fresh air and the moonroof acts as the outlet. When a third window (FR) is also open, we only observe a modest, incremental change in the aerosol concentration inside the cabin. Therefore, the case with two separate openings, RL and MR, is sufficient to establish a good ventilation inside the cabin. It may also be noted that the moonroof serves as a better air exit than the FR window considered by Mathai *et al.,*^1^ as it has a 25% larger open area as compared to the FR window, and also a lower pressure [Fig. 4(a)]. These features give rise to a 50% higher air exchange rate and lower aerosol concentrations when opening the moonroof instead of the FR window (comparing ACH for Configs. 3 and 5 in Table I). It is also possible that the vertical pattern of the airflow established by the open moonroof reduces cross-contamination and allows for the removal of airborne particles more effectively. Therefore, in vehicles with an active moonroof, Config. 5 can be considered to be a preferred driving configuration.

**FIG. 6.**
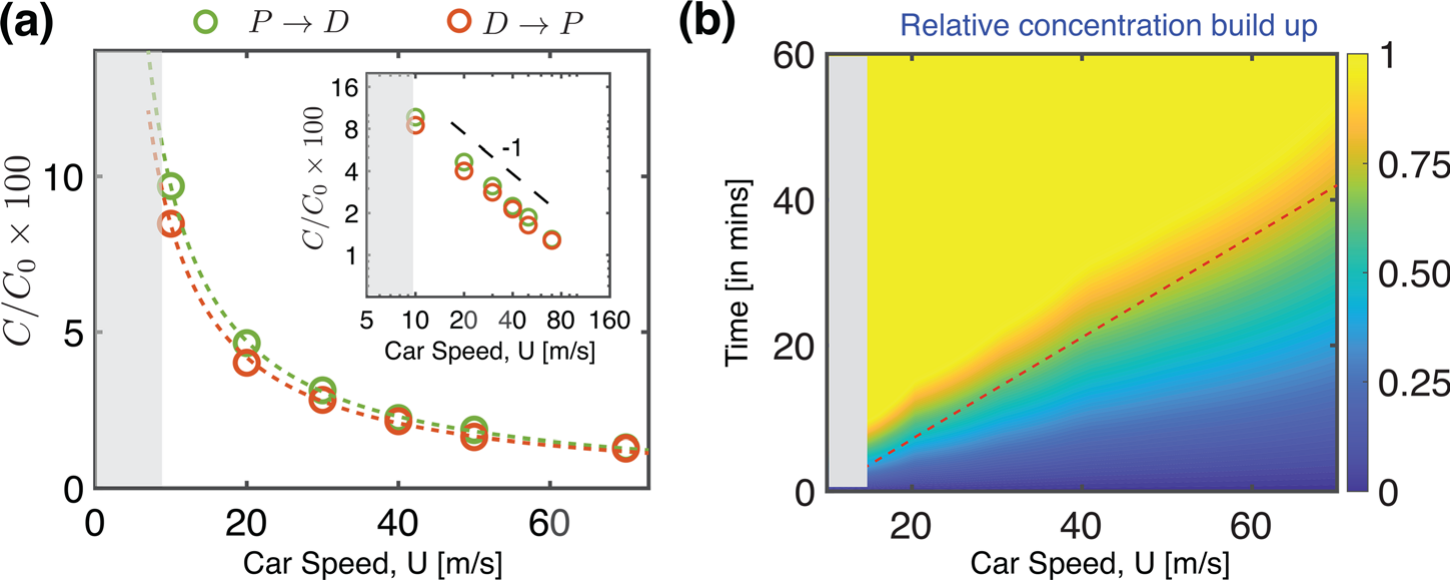
Influence of driving speed and ride duration on aerosol mode of transmission. (a) Relative concentration of aerosols in the cabin as a function of the car speed. This corresponds to a reference configuration wherein the rear left (RL) and front right (FR) windows are open. The green circles show the transmission from passenger to driver ( P→D), whereas the red circles show the transmission from driver to passenger ( D→P). The curves are fits to the data. Note that the *driver* is at a slightly higher risk when compared to the *passenger*, because of the rear-to-front circulating flow created by the two open windows (see also Ref. 1). The inset to (a) shows the same quantities plotted on a log –log scale. We observe a scaling exponent of −1, indicating that the overall flow patterns are unchanged over this range of speeds, and the average transport of aerosols is primarily a result of the advection, while the specific concentration patterns inside the cabin could be affected by the turbulent diffusion. (b) A *speed–time* diagram of the average concentration of aerosols inside the cabin. The infection risk may be thought to increase as the driving speed is lowered, and when the duration of ride increases. The red dashed line gives a relative measure of the infection risk, considering solely the aerosol mode of transmission, and following an approach similar to Yang *et al.*^29^ The upper limit of the color bar was arbitrary set, based on a few recommendations found in prior studies.^30,61^ The shaded region in gray represents the “low speed zone,” which is beyond the scope of the speed–time diagram presented here.

Table I presents a summary of the six configurations seen so far, with their corresponding ventilation rates and the average aerosol transport between the two occupants. All configurations presented in the table, as well as those reported by Mathai *et al.*^1^ are associated with a driving speed of 50 miles per hour. However, one can expect that the air change rate depends on the speed of the car, particularly given the fact that the ventilation flow is driven by the external pressure distribution which, assuming high Reynolds number, will scale with the square of the vehicle speed, *U*^2^. In many urban scenarios, a major share of driving occurs within city limits where it is uncommon to drive at a high speed. To look into these scenarios, we study the aerosol transmission patterns as a function of the driving speed. For convenience, we choose Config. 3 discussed in Mathai *et al.*^1^ and vary the driving speed. Figure 6(a) shows the variation of the concentration inside the cabin at different driving speeds. The green circular symbols correspond to the passenger-to-driver transmission, whereas the red circles show the driver-to-passenger concentration for the same case. As expected, the aerosol concentration increases with the lowering of car speed. The dashed lines show fits to the simulation data and the inset shows the same quantities plotted on a log–log scale. The scaling exponent of −1 confirms that the aerosol concentration scales inversely with speed, *U*, and suggests that the overall flow patterns are unchanged over the range of speeds considered here. Therefore, the average transport of aerosols is dominated by the advection term in Eq. (2), while the specific concentration patterns inside the cabin could be affected by the turbulent diffusion. Note that at very low speeds, the turbulence level may be considerably lower, and the flow patterns will likely be modified. The gray shaded region in Fig. 6 represents this low-speed zone where we do not extrapolate our results.

Recently, Yang *et al.*^29^ presented a space–time, social distancing guide of infection risk for two occupants in an enclosed environment. Their approach was based on the idea that the risk of infection reduces with increased social distancing, but rises with duration of the interaction. Therefore, every interaction could be expressed in terms of the physical separation (space) and duration of interaction (time), and assigned a risk (in a relative sense) using a space–time diagram. While this is widely applicable to a number of interactions that occur around us, the case of transportation needs to be considered slightly differently, mainly because the physical separation between occupants is limited inside the cabin of a passenger car and typically a (more-or-less) fixed distance. However, the driving speed has a crucial effect on the amount of aerosols circulating within the cabin, and thus the risk of transmission increases with the ride duration. These factors may be incorporated to generate an analogous speed–time diagram [Fig. 6(b)] to represent the relative infection risk when ride-sharing in a passenger car. The horizontal axis shows that with increasing driving speed the relative infection risk reduces and the vertical axis captures the increased risks due to drive duration. The upper limit of concentration here, corresponding to a drive duration of 60 min, has been arbitrarily chosen based on recent studies which suggest a high likelihood of infection for ride duration exceeding an hour.^30,61^ The diagonal line in Fig. 6(b) has been chosen based on the expectation that a ride duration shorter than 15 min at 50 mile per hour (22 m/s) may be considered relatively safe.^30^ Note that the above diagram considers only the aerosol mode of transmission wherein the expectation is that airborne particles follow the fluid flow and further, that the infection risk increases linearly with time. Further, we point out that the diagram presents only the relative risk, based on typical estimates in literature.^61,62^ When interpreting these, it is important to be aware of the biological variability in the virus shedding rates and susceptibilities of individuals.^63^

**FIG. 7.**
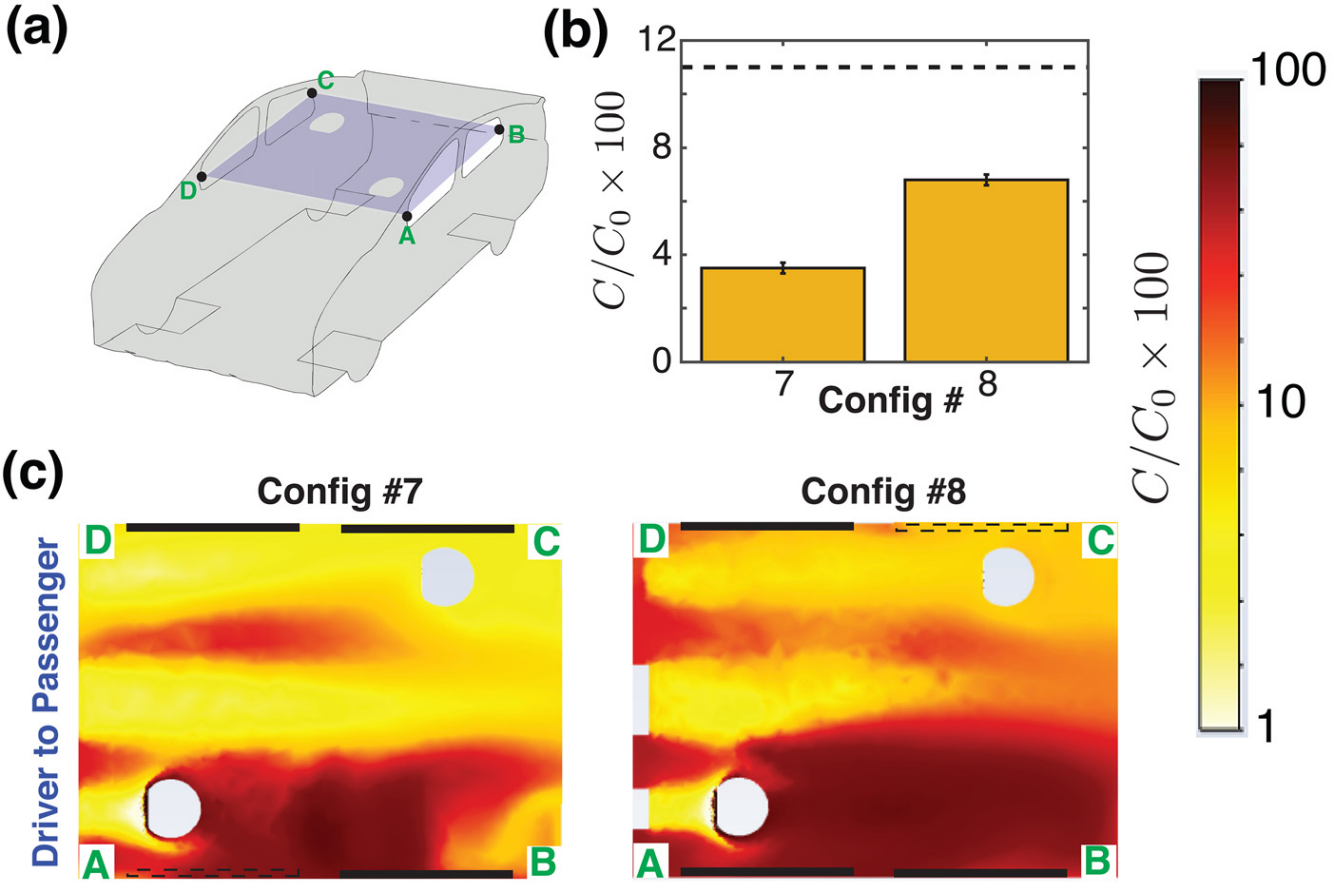
Driver-to-passenger transmission for a setting where the vehicle is not moving, as in city driving or when the vehicle is stuck in traffic (U = 0). (a) Schematic of the vehicle with a cut plane, A–B–C–D passing through the mid-height of the windows. (b) The bar graph shows the relative concentration of aerosols reaching the *passenger*, that originated from the *driver*. The dashed line gives the average concentration that reaches from the *driver* to the *passenger* when driving with all windows closed and air conditioning turned on (Config. 1 in Ref. 1). (c) Concentration field of the aerosols originating from the *driver* and reaching the *passenger*. Note that the line segment A–D is at the front of the car cabin, and the air flow speed U = 0 here.

Figure 6 recognizes the elevation of aerosol concentration when the driving speed is low, and therefore the configurations studied thus far (50 miles per hour driving speed) may not ensure a good ventilation within the cabin in settings where the driving speeds are well below 25 miles per hour (10 m/s). This can very well be the case in city traffic. To address this situation specifically, we consider a stationary passenger car with two occupants. In this situation, simply opening two windows or the moonroof would not be sufficient to create a good air flow pattern. Note that for U→0 the concentration $C/C_{o}\to 1$, somewhat unrealistically, as the transport due to molecular diffusion is the only remaining mechanism. In this situation, one could make use of the air conditioning flow in combination with open windows. Two configurations that might offer some benefit (Configs. 7 and 8) are shown in Fig. 7, which considers the aerosol transmission from the *driver* to the *passenger* (D → P). In Config. 7, the air conditioning vents at the front of the car serve as the primary entrance for fresh air entering the cabin, while air return vents at the rear of the cabin, along with the open front-left (FL) window besides the driver seat serve as the exits. Since the *driver* is simulated to be infected here (Fig. 7), the outward flow pattern established at the driver window allows to remove the high concentration of contaminated aerosols released by the *driver*. The average concentration of aerosols reaching the *passenger* remains within 4%, as shown in Fig. 7(b). In contrast, when the FL window is closed and the RR window besides the *passenger* is opened (Config. 8), an air path is created which drives the airborne particles from the infected *driver* toward the RR window, which leads to a slightly elevated concentration of aerosols ( ≈8%) near the *passenger* [Fig. 7(b)]. Even so, the concentration of aerosols in both configurations remain well below the case where all windows are closed [Fig. 7(b) dashed line].

**FIG. 8.**
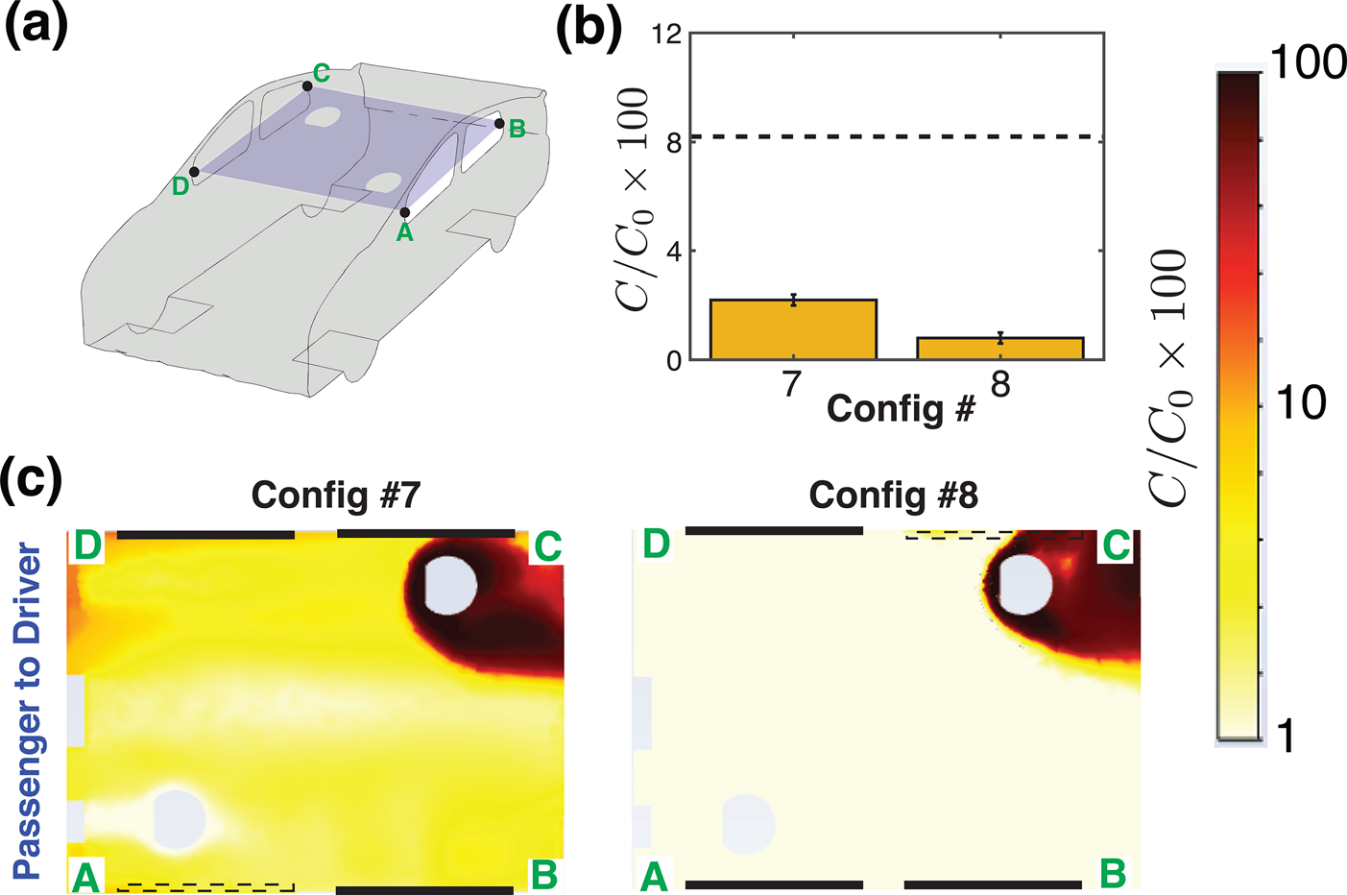
Passenger-to-driver transmission for a setting where the vehicle is not moving, as in city driving or when the vehicle is stuck in traffic (U = 0). (a) Schematic of the vehicle with a cut plane, A–B–C–D passing through the mid-height of the windows. (b) The bar graph shows the relative concentration of aerosols reaching the *driver*, that originated from the *passenger*. The dashed line gives the average concentration that reaches from the *passenger* to the *driver* when driving with all windows closed and air conditioning turned on (Config. 1 in Ref. 1). (c) Concentration field of the aerosols originating from the *passenger* and reaching the *driver*. Note that the line segment A–D is at the front of the car cabin, and the air flow speed U = 0 here.

Coming to the case of passenger-to-driver transmission (Fig. 8), the picture is more obvious. First, the front-to-rear flow path created by the air conditioning vents shields the *driver* from getting the aerosols released by the rear seat *passenger* in both configurations (Configs. 7 and 8). Second, when the window closest to the infected *passenger* is open (RR open, as in Config. 8), it serves as an exit, flushing out the high concentration of aerosols released by the *passenger* [Fig. 8(c)]. Both configurations have a relatively low average concentration of aerosols ( <3%). Combining the observations from Figs. 7 and 8, one may deduce that Config. 7, with air conditioning on and driver window open, might represent a good option for city driving and particularly when the vehicle is stuck in traffic.

## IV. CONCLUSIONS

We have performed a computational study employing Reynolds-averaged Navier–Stokes (RANS) simulations to investigate the overall flow fields and aerosol transmission patterns in a passenger car, extending the recent work by Mathai *et al.*^1^ to a wider variety of practically relevant driving scenarios. Although the key conclusions are still somewhat expected—more ventilation means lower aerosol concentrations and lower pathogenic transmission risks—there are nevertheless several less-obvious conclusions to be drawn. We have identified that when driving at high speeds (50 miles per hour or 22 m/s), partially opening windows might be sufficient to remove potentially pathogenic airborne particles from the cabin. This provides a practical compromise when having to drive under poor weather conditions. Additionally, our analysis has shown the utility of opening the moonroof on vehicles while driving, as it serves as an unimpeded exit for the contaminated cabin air. The results also emphasize a point made by Mathai *et al.*^1^ that the microclimate—the distribution of aerosols within the cabin—is as important as the more integral measures of ventilation, such as the air changes per hour (ACH) when considering the occupants' health risks.

At lower driving speeds, covering the range of 10–22 m/s, we find that the aerosol concentration can be elevated, scaling inversely with the vehicle speed. We have presented a speed–time diagram which allows for an assessment of the relative risk of aerosol transmission as a function of the driving speed and ride duration. Finally, we considered the case where the vehicle is stuck in traffic, in which case it may be beneficial to open the front-left window adjacent to the *driver*, while leaving the air conditioning at full blast. The analyses have shown that this configuration presents a good compromise for removal of the aerosols released by both the *driver* and the rear seat *passenger*.

Several of the assessments made in the present study were based on existing general guidelines to evaluate indoor air quality and ventilation rates.^27,29^ We note that our analyses are strictly limited to the airborne mode of transmission. Other modes of transmission, such as those due to violent respiratory expulsions^64,65^ that occur during coughing or sneezing or loud speech, are not encompassed in the present study. Furthermore, the steady RANS approach adopted here presents a simplified, time-averaged treatment of the unsteady Navier–Stokes equations that govern the fluid flow. Future studies employing more accurate computational methods, such as large eddy simulations (LES), fully resolved direct numerical simulations (DNS), or field testing, could yield better insights with more accurate assessment of the flow patterns and species transport.

## Data Availability

The data that support the findings of this study are available from the corresponding author upon reasonable request.

## References

[1] V. Mathai , A. Das , J. A. Bailey , and K. Breuer , “ Airflows inside passenger cars and implications for airborne disease transmission,” Sci. Adv. 7(1), eabe0166 (2021). 10.1126/sciadv.abe0166 33277325 PMC7775778

[2] L. Morawska , J. W. Tang , W. Bahnfleth , P. M. Bluyssen , A. Boerstra , G. Buonanno *et al.*, “ How can airborne transmission of COVID-19 indoors be minimised?,” Environ. Int. 142, 105832 (2020). 10.1016/j.envint.2020.105832 32521345 PMC7250761

[3] P. Bahl , C. Doolan , C. de Silva , A. A. Chughtai , L. Bourouiba , and C. R. MacIntyre , “ Airborne or droplet precautions for health workers treating COVID-19?,” J. Infect. Dis. (published online) (2020). 10.1093/infdis/jiaa189 PMC7184471 32301491

[4] T. Greenhalgh , J. L. Jimenez , K. A. Prather , Z. Tufekci , D. Fisman , and R. Schooley , “ Ten scientific reasons in support of airborne transmission of SARS-CoV-2,” Lancet 397(10285), 1603–1605 (2021). 10.1016/S0140-6736(21)00869-2 33865497 PMC8049599

[5] J. W. Tang , L. C. Marr , Y. Li , and S. J. Dancer , “ Covid-19 has redefined airborne transmission,” BMJ 373, n913 (2021). 10.1136/bmj.n913 33853842

[6] M. Klompas , M. A. Baker , and C. Rhee , “ Airborne transmission of SARS-CoV-2: Theoretical considerations and available evidence,” JAMA 324, 441 (2020). 10.1001/jama.2020.12458 32749495

[7] V. Stadnytskyi , C. E. Bax , A. Bax , and P. Anfinrud , “ The airborne lifetime of small speech droplets and their potential importance in SARS-CoV-2 transmission,” Proc. Natl. Acad. Sci. 117(22), 11875–11877 (2020). 10.1073/pnas.2006874117 32404416 PMC7275719

[8] M. Z. Bazant and J. W. Bush , “ A guideline to limit indoor airborne transmission of COVID-19,” Proc. Natl. Acad. Sci. 118(17), e2018995118 (2021). 10.1073/pnas.2018995118 33858987 PMC8092463

[9] S. Asadi , A. S. Wexler , C. D. Cappa , S. Barreda , N. M. Bouvier , and W. D. Ristenpart , “ Effect of voicing and articulation manner on aerosol particle emission during human speech,” PLoS One 15(1), e0227699 (2020). 10.1371/journal.pone.0227699 31986165 PMC6984704

[10] L. Bourouiba , E. Dehandschoewercker , and J. W. Bush , “ Violent expiratory events: On coughing and sneezing,” J. Fluid Mech. 745, 537–563 (2014). 10.1017/jfm.2014.88

[11] G. R. Johnson , L. Morawska , Z. D. Ristovski , M. Hargreaves , K. Mengersen , C. Y. Hang Chao , M. P. Wan , Y. Li , X. Xie , D. Katoshevski *et al.*, “ Modality of human expired aerosol size distributions,” J. Aerosol Sci. 42(12), 839–851 (2011). 10.1016/j.jaerosci.2011.07.009 PMC7126899 32287373

[12] J. P. Duguid , “ The size and the duration of air-carriage of respiratory droplets and droplet-nuclei,” Epidemiol. Infect. 44(6), 471–479 (1946). 10.1017/S0022172400019288 PMC2234804 20475760

[13] W. F. Wells *et al.*, “ On air-borne infection. Study II. Droplets and droplet nuclei,” Am. J. Hyg. 20, 611–618 (1934). 10.1093/oxfordjournals.aje.a118097

[14] R. Mittal , R. Ni , and J.-H. Seo , “ The flow physics of COVID-19,” J. Fluid Mech. 894, F2 (2020). 10.1017/jfm.2020.330

[15] R. Wölfel , V. M. Corman , W. Guggemos , M. Seilmaier , S. Zange *et al.*, “ Virological assessment of hospitalized patients with COVID-2019,” Nature 581(7809), 465–469 (2020). 10.1038/s41586-020-2196-x 32235945

[16] S.-A. Lee , S. A. Grinshpun , and T. Reponen , “ Respiratory performance offered by N95 respirators and surgical masks: Human subject evaluation with NaCl aerosol representing bacterial and viral particle size range,” Ann. Occup. Hyg. 52(3), 177–185 (2008). 10.1093/annhyg/men005 18326870 PMC7539566

[17] N. H. L. Leung , D. K. W. Chu , E. Y. C. Shiu , K.-H. Chan , J. J. McDevitt *et al.*, “ Respiratory virus shedding in exhaled breath and efficacy of face masks,” Nat. Med. 26(5), 676–680 (2020). 10.1038/s41591-020-0843-2 32371934 PMC8238571

[18] M. Meselson , “ Droplets and aerosols in the transmission of SARS-CoV-2,” N. Engl. J. Med. 382(21), 2063–2063 (2020). 10.1056/NEJMc2009324 32294374 PMC7179963

[19] G. Vaidyanathan , “ Coronavirus variants are spreading in India–What scientists know so far,” Nature 593(7859), 321–322 (2021). 10.1038/d41586-021-01274-7 33976409

[20] Y.-T. Li , T.-C. Chen , S.-Y. Lin , M. Mase , S. Murakami , T. Horimoto , and H.-W. Chen , “ Emerging lethal infectious bronchitis coronavirus variants with multiorgan tropism,” Transboundary Emerging Dis. 67(2), 884–893 (2020). 10.1111/tbed.13412 PMC7138078 31682070

[21] R. Zhang , Y. Li , A. L. Zhang , Y. Wang , and M. J. Molina , “ Identifying airborne transmission as the dominant route for the spread of COVID-19,” Proc. Natl. Acad. Sci. 117(26), 14857 (2020). 10.1073/pnas.2009637117 32527856 PMC7334447

[22] J. K. Gupta , C.-H. Lin , and Q. Chen , “ Characterizing exhaled airflow from breathing and talking,” Indoor air 20(1), 31–39 (2010). 10.1111/j.1600-0668.2009.00623.x 20028433

[23] L. Bourouiba , “ Turbulent gas clouds and respiratory pathogen emissions: Potential implications for reducing transmission of COVID-19,” JAMA 323(18), 1837–1838 (2020). 10.1001/jama.2020.4756 32215590

[24] Y. Liu , Z. Ning , Y. Chen , M. Guo , Y. Liu , N. Kumar Gali , L. Sun , Y. Duan , J. Cai , D. Westerdahl *et al.*, “ Aerodynamic analysis of SARS-CoV-2 in two Wuhan hospitals,” Nature 582(7813), 557–560 (2020). 10.1038/s41586-020-2271-3 32340022

[25] G. A. Somsen , C. van Rijn , S. Kooij , R. A. Bem , and D. Bonn , “ Small droplet aerosols in poorly ventilated spaces and SARS-CoV-2 transmission,” Lancet Respir. Med. 8(7), 658 (2020). 10.1016/S2213-2600(20)30245-9 32473123 PMC7255254

[26] R. K. Bhagat and P. F. Linden , “ Displacement ventilation: A viable ventilation strategy for makeshift hospitals and public buildings to contain COVID-19 and other airborne diseases,” R. Soc. Open Sci. 7(9), 200680 (2020). 10.1098/rsos.200680 33047029 PMC7540764

[27] P. A. Jensen , L. A. Lambert , M. F. Iademarco , and R. Ridzon , “ Guidelines for preventing the transmission of mycobacterium tuberculosis in health-care settings,” Report No. 54 (RR17), 1–141 (2005). 16382216

[28] P. F. Linden , “ The fluid mechanics of natural ventilation,” Annu. Rev. Fluid Mech. 31(1), 201–238 (1999). 10.1146/annurev.fluid.31.1.201

[29] F. Yang , A. A. Pahlavan , S. Mendez , M. Abkarian , and H. A. Stone , “ Towards improved social distancing guidelines: Space and time dependence of virus transmission from speech-driven aerosol transport between two individuals,” Phys. Rev. Fluids 5(12), 122501 (2020). 10.1103/PhysRevFluids.5.122501

[30] J. Allen , J. Spengler , and R. Corsi , *Is There Coronavirus in Your Car? Here's How You Can Protect Yourself* ( USA Today, 2020).

[31] T. Li , L. Rong , and A. Zhang , “ Assessing regional risk of COVID-19 infection from Wuhan via high-speed rail,” Transp. Policy 106, 226–238 (2021). 10.1016/j.tranpol.2021.04.009 PMC8043780 33867701

[32] Z. Zhang , T. Han , K. H. Yoo , J. Capecelatro , A. L. Boehman , and K. Maki , “ Disease transmission through expiratory aerosols on an urban bus,” Phys. Fluids 33(1), 015116 (2021). 10.1063/5.0037452 PMC7976046 33746484

[33] N. J. Edwards , R. Widrick , J. Wilmes , B. Breisch , M. Gerschefske , J. Sullivan , R. Potember , and A. Espinoza-Calvio , “ Reducing COVID-19 airborne transmission risks on public transportation buses: An empirical study on aerosol dispersion and control,” medRxiv (2021).

[34] Y. Wong , “ To limit coronavirus risks on public transport, here's what we can learn from efforts overseas,” The Conversation (2020).

[35] R. Pombal , I. Hosegood , and D. Powell , “ Risk of COVID-19 during air travel,” JAMA 324(17), 1798–1798 (2020). 10.1001/jama.2020.19108 33022035

[36] See N. Rorres , https://www.rtd-denver.com/sites/default/files/files/2020-10/Airflow%20Through%20Buses.pdf for “ Airflow through buses, Regional Transportation District (RTD), Denver, CO” (2020).

[37] L. D. Knibbs , L. Morawska , and S. C. Bell , “ The risk of airborne influenza transmission in passenger cars,” Epidemiol. Infect. 140(3), 474–478 (2012). 10.1017/S0950268811000835 21733264

[38] B. Fletcher and C. J. Saunders , “ Air change rates in stationary and moving motor vehicles,” J. Hazard. Mater. 38(2), 243–256 (1994). 10.1016/0304-3894(94)90026-4

[39] W. Ott , N. Klepeis , and P. Switzer , “ Air change rates of motor vehicles and in-vehicle pollutant concentrations from secondhand smoke,” J. Exposure Sci. Environ. Epidemiol. 18(3), 312–325 (2008). 10.1038/sj.jes.7500601 17637707

[40] E. M. Saber and M. Bazargan , “ Dynamic behavior modeling of cigarette smoke particles inside the car cabin with different ventilation scenarios,” Int. J. Environ. Sci. Technol. 8(4), 747–764 (2011). 10.1007/BF03326259

[41] D. Müller , D. Klingelhöfer , S. Uibel , and D. A. Groneberg , “ Car indoor air pollution—Analysis of potential sources,” J. Occup. Med. Toxicol. 6(1), 33 (2011). 10.1186/1745-6673-6-33 22177291 PMC3261090

[42] Y. He , Q. Zhang , C. An , Y. Wang , Z. Xu , and Z. Zhang , “ Computational investigation and passive control of vehicle sunroof buffeting,” J. Vib. Control 26(9–10), 747–756 (2020). 10.1177/1077546319889784

[43] S. Yin , Z. Gu , Y. Zong , L. Zheng , Z. Yang , and T. Huang , “ Sound quality evaluation of automobile side-window buffeting noise based on large-eddy simulation,” J. Low Frequency Noise, Vib. Active Control 38(2), 207–223 (2019). 10.1177/1461348418816268

[44] V. Mathai , “ The air we breathe in a car,” Phys. Today 74(6), 66–67 (2021). 10.1063/PT.3.4779

[45] D. A. Edwards , D. Ausiello , J. Salzman , T. Devlin , R. Langer , B. J. Beddingfield , A. C. Fears , L. A. Doyle-Meyers , R. K. Redmann , S. Z. Killeen *et al.*, “ Exhaled aerosol increases with COVID-19 infection, age, and obesity,” Proc. Natl. Acad. Sci. 118(8), e2021830118 (2021). 10.1073/pnas.2021830118 33563754 PMC7923364

[46] S. Khatoon and M.-H. Kim , “ Thermal comfort in the passenger compartment using a 3-D numerical analysis and comparison with Fanger's comfort models,” Energies 13(3), 690 (2020). 10.3390/en13030690

[47] S. V. Patankar , *Numerical Heat Transfer and Fluid Flow* ( Hemisphere Publishing Corp., Washington, DC, 1980).

[48] S. B. Pope , *Turbulent Flows* (Cambridge University Press, 2001).

[49] S. Stanojevic , M. Ponjavic , S. Stanojevic , A. Stevanovic , and S. Radojicic , “ Simulation and prediction of spread of COVID-19 in the Republic of Serbia by SEAIHRDS model of disease transmission,” Microb. Risk Anal. 18, 100161 (2021). 10.1016/j.mran.2021.100161 33723516 PMC7946545

[50] E. Furbo , *Evaluation of RANS Turbulence Models for Flow Problems with Significant Impact of Boundary Layers* (Uppsala University, Sweden, 2010).

[51] W. C. Reynolds , “ Computation of turbulent flows,” Annu. Rev. Fluid Mech. 8(1), 183–208 (1976). 10.1146/annurev.fl.08.010176.001151

[52] D. A. Jones , M. Chapuis , M. Liefvendahl , D. Norrison , and R. Widjaja , “ RANS simulations using OpenFOAM software,” Report No. 2012/1175723 ( Defence Science and Technology Group, Fishermans Bend, Victoria, Australia, 2016).

[53] K. Talaat , M. Abuhegazy , O. A. Mahfoze , O. Anderoglu , and S. V. Poroseva , “ Simulation of aerosol transmission on a Boeing 737 airplane with intervention measures for COVID-19 mitigation,” Phys. Fluids 33(3), 033312 (2021). 10.1063/5.0044720 PMC8060968 33897238

[54] C. C. Ooi , A. Suwardi , Z. L. Ou Yang , G. Xu , C. K. I. Tan , D. Daniel , H. Li , Z. Ge , F. Y. Leong , K. Marimuthu *et al.*, “ Risk assessment of airborne COVID-19 exposure in social settings,” Phys. Fluids 33(8), 087118 (2021). 10.1063/5.0055547 PMC8450907 34552314

[55] R. Hetherington , A. B. M. Toufique Hasan , A. Khan , D. Roy , M. Salehin , and Z. Wadud , “ Exposure risk analysis of COVID-19 for a ride-sharing motorbike taxi,” Phys. Fluids 33(11), 113319 (2021). 10.1063/5.0069454 PMC8726634 35002199

[56] P. Dey , S. K. Saha , and S. Sarkar , “ Study of the interactions of sneezing droplets with particulate matter in a polluted environment,” Phys. Fluids 33(11), 113310 (2021). 10.1063/5.0067517 PMC8597716 34803363

[57] S. Kumar and H. P. Lee , “ The perspective of fluid flow behavior of respiratory droplets and aerosols through the facemasks in context of SARS-CoV-2,” Phys. Fluids 32(11), 111301 (2020). 10.1063/5.0029767 PMC7713871 33281434

[58] Q. Chen and N.-T. Chao , “ Comparing turbulence models for buoyant plume and displacement ventilation simulation,” Indoor Built. Environ. 6(3), 140–149 (1997). 10.1177/1420326X9700600304

[59] R. Rossi and G. Iaccarino , “ Numerical analysis and modeling of plume meandering in passive scalar dispersion downstream of a wall-mounted cube,” Int. J. Heat Fluid Flow 43, 137–148 (2013). 10.1016/j.ijheatfluidflow.2013.04.006

[60] Z. Yang , Z. Gu , J. Tu , G. Dong , and Y. Wang , “ Numerical analysis and passive control of a car side window buffeting noise based on scale-adaptive simulation,” Appl. Acoust. 79, 23–34 (2014). 10.1016/j.apacoust.2013.12.006

[61] J. A. Lednicky , M. Lauzardo , M. M. Alam , M. A. Elbadry , C. J. Stephenson , J. C. Gibson , and J. G. Morris, Jr. , “ Isolation of SARS-CoV-2 from the air in a car driven by a COVID patient with mild illness,” Int. J. Infect. Dis. 108, 212 (2021). 10.1016/j.ijid.2021.04.063 33901650 PMC8064821

[62] W. Yang , S. Elankumaran , and L. C. Marr , “ Concentrations and size distributions of airborne influenza a viruses measured indoors at a health centre, a day-care centre and on aeroplanes,” J. R. Soc. Interface 8(61), 1176–1184 (2011). 10.1098/rsif.2010.0686 21300628 PMC3119883

[63] T. C. Jones , B. Mühlemann , T. Veith , G. Biele , M. Zuchowski , J. Hoffmann , A. Stein , A. Edelmann , V. M. Corman , and C. Drosten , “ An analysis of SARS-CoV-2 viral load by patient age,” MedRxiv (2020). 10.1101/2020.06.08.20125484

[64] K. L. Chong , C. S. Ng , N. Hori , R. Yang , R. Verzicco , and D. Lohse , “ Extended lifetime of respiratory droplets in a turbulent vapor puff and its implications on airborne disease transmission,” Phys. Rev. Lett. 126(3), 034502 (2021). 10.1103/PhysRevLett.126.034502 33543958

[65] M. Abkarian , S. Mendez , N. Xue , F. Yang , and H. A. Stone , “ Speech can produce jet-like transport relevant to asymptomatic spreading of virus,” Proc. Natl. Acad. Sci. 117(41), 25237–25245 (2020). 10.1073/pnas.2012156117 32978297 PMC7568291

